# *Microcystis* bloom control using hydrogen peroxide and floating sodium percarbonate algaecide Lake Guard Oxy in Florida

**DOI:** 10.1128/aem.01950-25

**Published:** 2025-12-02

**Authors:** Taylor L. Hancock, Elizabeth K. Dahedl, Hidetoshi Urakawa

**Affiliations:** 1 Department of Biology, Doane University 2724, Crete, Nebraska, USA; 2 Department of Ecology and Environmental Studies, The Water School, Florida Gulf Coast University 648135, Fort Myers, Florida, USA; 3 School of Geosciences, University of South Florida 638270, Tampa, Florida, USA; Michigan State University, East Lansing, Michigan, USA

**Keywords:** metatranscriptome, cyanobacteria, HAB, mitigation, nitrogen fixation

## Abstract

Harmful algal blooms (HABs) pose a critical threat to aquatic ecosystems, human health, and economies globally, necessitating effective mitigation strategies. This study investigated the efficacy of two hydrogen peroxide treatments, liquid hydrogen peroxide and sodium percarbonate algaecide Lake Guard Oxy (LGOxy), in the field during a *Microcystis* bloom in the Caloosahatchee River, Florida, USA. Initial results indicated bloom suppression following both the liquid hydrogen peroxide and LGOxy treatments as 79% and 46% reduction in cyanobacterial relative abundance in 16S rRNA gene amplicon sequencing, 81% and 90% reduction in chlorophyll-a, and 91% and 97% reduction in *Microcystis* colony density. Both treatments were effective in reducing cyanobacterial abundance in the short term, from immediately after application to the following day. However, their effects did not persist longer term (several days to a week), likely because water exchange occurred through the connection to a flowing system. LGOxy exhibited a more targeted impact on *Microcystis* than other cyanobacterial species, likely because *Microcystis* forms thin surface blooms that remain in contact with the buoyant LGOxy particles. We also observed increased upregulation of the expression of nitrogen fixation genes in diazotrophs, such as *Dolichospermum*, based on transcriptomic analysis, particularly following LGOxy application. Ultimately, three treatments within 2 weeks (one liquid application and two LGOxy treatments) were not sufficient to fully eliminate the cHAB from the riverine study site after 14 days. This underscores the limitations of hydrogen peroxide-based treatments when target HABs are already established and the challenges facing deployment in open or actively flowing hydrologic systems.

**IMPORTANCE:**

Harmful algal blooms (HABs) pose a significant threat to aquatic ecosystems, public health, and global economies, necessitating the development of effective mitigation strategies. Hydrogen peroxide has emerged as an environmentally friendly algaecide suitable for large-scale algal bloom control. Traditionally, hydrogen peroxide has been applied in liquid form; however, solid peroxide formulations are increasingly preferred by water managers due to their advantages in storage, transport, and handling safety. Despite these practical benefits, a direct comparison of the efficacy of liquid versus solid hydrogen peroxide formulations for algal bloom mitigation remains limited. This case study evaluated the effectiveness of two hydrogen peroxide-based treatments, liquid hydrogen peroxide and Lake Guard Oxy, during a field application targeting a *Microcystis* bloom in the Caloosahatchee River, Florida, USA.

## INTRODUCTION

Harmful algal blooms (HABs) present a significant global challenge to water quality, affecting ecosystem structure and function, human health, and local recreational and tourist-based economies (1, 2). Effective management of HABs requires both long-term mitigation strategies and immediate actions (3). Nutrient control is essential for long-term water management and mitigation practices as it addresses the root cause of nutrient-driven HABs. However, once blooms occur, short-term solutions are necessary to mitigate their immediate negative impacts (4).

Among the various methods proposed and tested for the treatment of HABs, application of hydrogen peroxide has emerged as an environmentally friendly and cost-effective option (5–7). The body of knowledge surrounding this treatment method is rapidly expanding, largely driven by mesocosm studies (6, 8–10). Despite these advances, there is a growing need for evaluating *in situ* field applications to better understand the practical challenges and effectiveness of hydrogen peroxide treatments in natural water bodies (5, 11–13).

Field applications of hydrogen peroxide are particularly challenging because their effectiveness is influenced by climatic and hydrological conditions. Environmental factors, such as concentrations of dissolved metals (e.g., copper, iron, and manganese) and organic matter, can also alter the decay rate of hydrogen peroxide (14), affecting treatment outcomes. The success of these treatments can vary widely based on these conditions and the strategies employed, with documented cases ranging from successful mitigation to complete failure (9, 12, 13, 15).

Southern Florida is a region particularly susceptible to HABs, with its tropical/subtropical climate and expanding anthropogenic nutrient loading providing ideal conditions for the growth of cyanobacteria, even during the winter months (16). In May of 2021, the Caloosahatchee River in Florida experienced a HAB dominated by *Microcystis aeruginosa* (16). The Lee County (Florida) weekly HAB monitoring survey, which uses the index system described in Urakawa et al. (17), recorded this HAB as level 3 (abundant: accumulation, high cell concentrations, clumps, or streaks). During this period, the water was treated by two separate parties, Florida Gulf Coast University (FGCU) and BlueGreen Water Technologies (Modi’in-Maccabim-Re’ut, Israel), using regular liquid hydrogen peroxide and granulated Lake Guard Oxy, respectively, over the course of 2 weeks. This case study documented the effects of these different hydrogen peroxide-based treatments, providing valuable chemical and biological data to better understand cHAB mitigation techniques. The findings from this case study offer insights that are useful for water managers and scientists in addressing the challenges of mitigating cHABs.

## RESULTS AND DISCUSSION

### Field site and hydrogen peroxide treatment design

**Fig 1:**
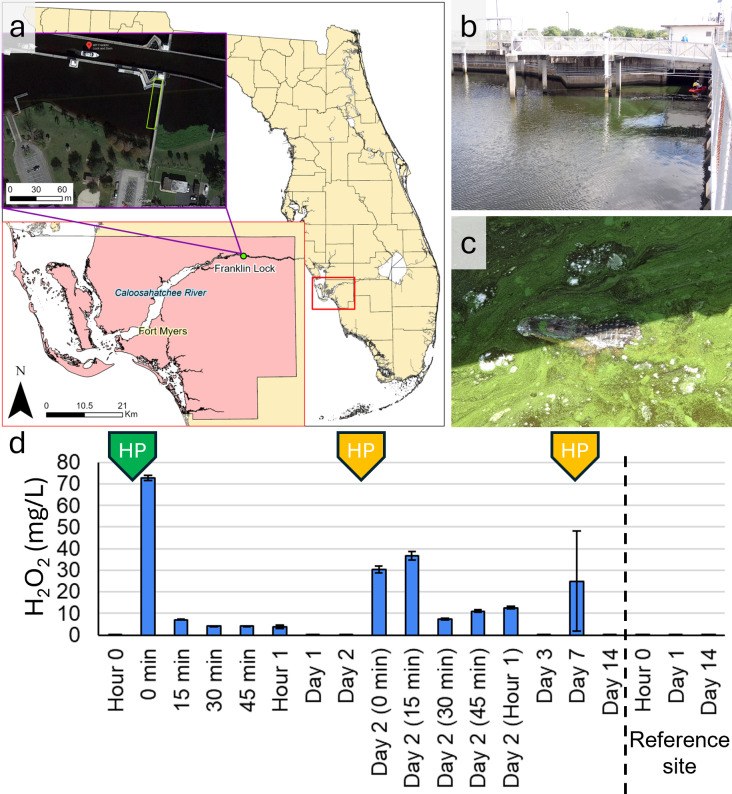
Sampling site and hydrogen peroxide concentrations. (**a**) Franklin Lock and Dam (S-79) on the Caloosahatchee River, FL, USA. The maps were created in ArcGIS Pro, using data from the Southwest Florida Water Management District. (**b**) Treatment area on the downstream side of the lock. The depth of water was approximately 3.5 m. (**c**) *Microcystis aeruginosa* scum, Lake Guard Oxy and alligator. Lime green dense algal cells in surface water forming a bloom prior to treatment on 25 May 2021. Wildlife was present in the treatment area, but no mortality or injury was observed. (**d**) Hydrogen peroxide concentrations before and after liquid hydrogen peroxide spray (green) and Lake Guard oxy granular (yellow) treatments.

On 25 May 2021, a bloom of *Microcystis* at Franklin Lock and Dam (S-79) on the Caloosahatchee River, FL, USA, was observed during routine water quality monitoring (16). This prompted a field treatment trial using hydrogen peroxide. We selected a semi-enclosed, sheltered area adjacent to the lock structure for treatment as it does not experience constant river flow but is influenced by a daily tidal cycle (Fig. 1). Hydrogen peroxide treatments were repeated three times on the same parcel of water: the first was conducted by FGCU using liquid hydrogen peroxide; the second and third applications were performed by BlueGreen Water Technologies using Lake Guard Oxy, a hydrogen peroxide-based algaecide composed of 98% (wt/wt) sodium percarbonate that releases hydrogen peroxide and 2% (wt/wt) of an inert biodegradable encapsulating agent that floats and time-releases the active ingredient on the water surface. In the first treatment, 3% hydrogen peroxide solution was directly applied from a kayak to the surface cyanobacterial scum via a 1-gallon hand pump sprayer in a 400 m^2^ area (8 m x 50 m) against the downstream side of the lock structure (3.5 m in depth), for a final theoretical concentration of 20 mg/L (580 µM, 0.0018%) based on 10 cm of depth. The second and third treatments were performed on days 2 and 7, respectively, by BlueGreen Water Technologies using a motorized boat. Sixty-five kilograms (143.3 pounds) of Lake Guard Oxy was applied to approximately 2,023 m^2^ (0.5 acres) (Fig. 1). This concentration is equivalent to the maximum single treatment dose of Lake Guard Oxy provided on the US EPA approved label, which is recommended to use when Chl-a concentration exceeds 50 µg/L or cyanobacterial cell density is more than 10^5^ cells/L. The effects of these treatments were determined by sampling the surface water (< 30 cm depth) before treatment and 1 h after the first treatment, and on days 1, 2, 3, 7, and 14 at 2 pm EST. The day 2 treatment was conducted after the sampling of FGCU on that day, and the day 7 sample was collected after 1 hour post-treatment. Physiochemical parameters (dissolved oxygen (DO), specific conductance, and pH) were measured in triplicate with an Aqua TROLL sonde (In-Situ, CO, USA) at three locations equally spaced across the length of our treatment area. We also sampled a nearby area outside of the treatment area on days 0, 1, and 14 to serve as a reference site.

### Hydrogen peroxide dynamics and impact on wildlife

Hydrogen peroxide in surface water prior to the liquid hydrogen peroxide treatment was 0.052 mg/L (1.5 µM), which falls within the range of previously documented concentrations used in the Caloosahatchee River (17, 18). Despite our 20 mg/L (580 µM) theoretical concentration, the highest hydrogen peroxide concentration measured in this experiment immediately after treatment (0 min) was 73.7 mg/L (2,137 µM), which then quickly decreased to 7.1 mg/L (208 µM) after 15 min and then 4.3 mg/L (125 µM) after 1 h due to dilution and degradation in the open aquatic environment (Fig. 1). The concentration returned to background level by the next day (after 24 h, 0.014 mg/L [0.41 µM]) (Fig. 1).

The 15 min interval hydrogen peroxide concentration sampling conducted immediately after Lake Guard Oxy application revealed a different profile compared to that of the liquid hydrogen peroxide application following its respective treatment (Fig. 1d). The peak concentration occurred 15 min after treatment (37.3 mg/L [1,082 µM]), rather than immediately upon application. We assumed that this was caused by the gradual release of hydrogen peroxide from sodium carbonate peroxyhydrate. Additionally, while the maximum concentration measured was lower than the liquid application, elevated concentrations persisted longer and did not quickly decrease as observed in the liquid application, with 12.6 mg/L (370 µM) measured after 1 h, and the concentration returned to the background level by the next day (0.04 mg/L [1.3 µM]). For the Lake Guard Oxy application on day 7, we measured 24.9 mg/L (733 µM) after 1 hour post-treatment. Differences in hydrogen peroxide concentrations shortly after application were attributed to the treatment chemical formulations; liquid hydrogen peroxide dispersed rapidly and degraded quickly in the aquatic environment, whereas Lake Guard Oxy released hydrogen peroxide more gradually, although the release was still largely completed within a day.

The impact of hydrogen peroxide on wildlife was examined visually by walking along the shore to the end of the enclosed area and walking on the lock structure to note the presence or absence of wildlife (reptiles, fish, birds, and mammals) mortality during each sampling period. While the biological responses of hydrogen peroxide treatments to wildlife were not quantified, we monitored environmental conditions around the treatment area. We found American alligators (*Alligator mississippiensis*) (Fig. 1), West Indian manatees (*Trichechus manatus*), and a variety of birds and fish inhabiting the treatment area during our sampling appeared unaffected, with no mortalities observed after treatment exposure at our sampling site within 14 days. This is expected as the concentration needed for macrofauna toxicity is much higher than our treatment concentration, with 1 hour of continuous exposure in ≥3,401 mg/L (100 mM) hydrogen peroxide required to incur limited mortality in some fingerling fishes (19, 20). However, it does not mean that microscopic wildlife like zooplankton were unaffected. We did not survey these communities; however, since the 48 hour LC50 concentration for zooplankton can be as low as 1.7 mg/L (50 µM), the treatment concentrations used likely had a negative impact (21).

### Cyanobacterial responses to the hydrogen peroxide treatments

**Fig 2:**
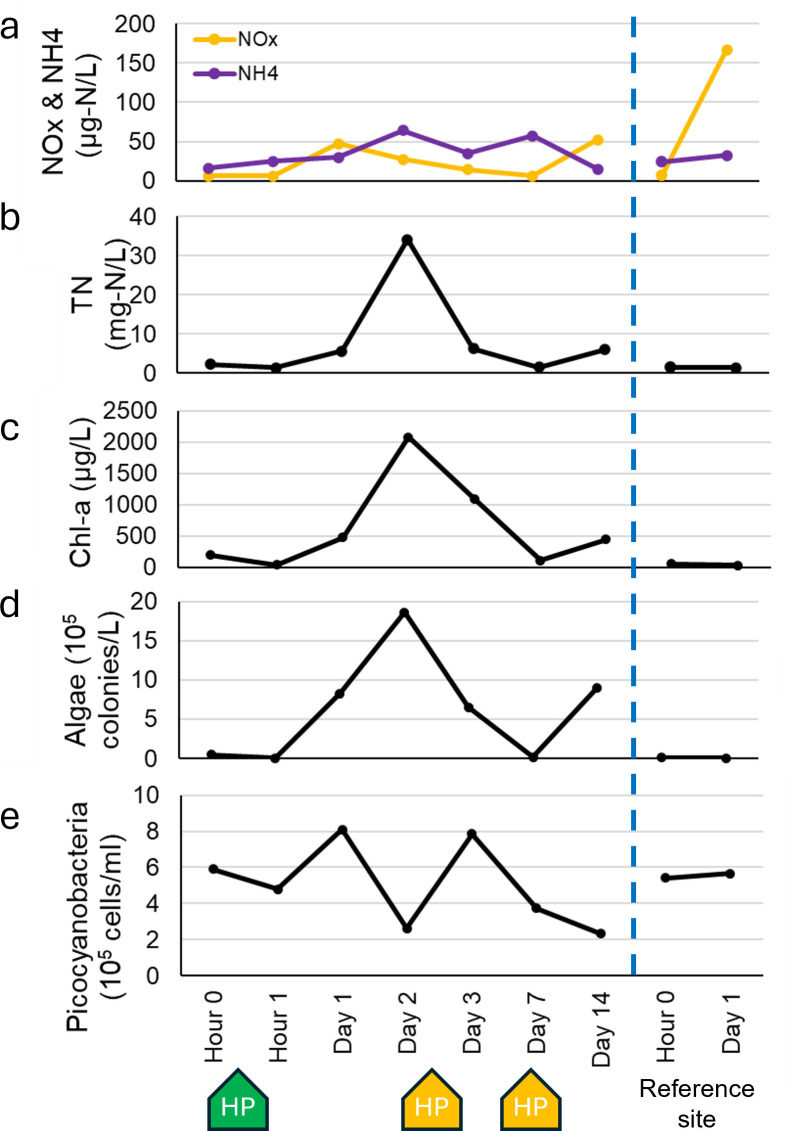
Time series of nitrate/nitrite and ammonium (**a**), total nitrogen (**b**), Chl-a (**c**), algal colony density (**d**), and picocyanobacterial cell density (**e**). Liquid hydrogen peroxide spray (green) and Lake Guard Oxy granular (yellow) treatment additions denoted as markers.

Similar to a previous field application study conducted at this site earlier the same year (12), the most dramatic response occurred within hours of treatment. Within 1 hour of the initial liquid hydrogen peroxide treatment (1 h after on day 1), Chl-a and algal colony density decreased by 81.2% and 91.3%, respectively (Fig. 2). During this period, the initial liquid hydrogen peroxide treatment (hour 1) reduced cyanobacteria sequences in the bacterial community by 79%. After treatment, total microcystin levels declined from 3.7 to 2.4 µg/L, accompanied by an increase in the extracellular fraction from 57% to 79% (Table 1).

**TABLE 1:** Water quality parameters^*a*^

Type	Time	Temp (°C)	DO (%)	Specific conductivity (µS/cm)	pH	Total microcystin (µg/L)	Extracellular microcystin (µg/L)	CDOM (µg/L)
Treatment	Hour 0	28	98	1,853	8.2	3.7	2.1	208
	Hour 1	28	97	1,779	8.1	2.4	1.9	237
	Day 1	27	91	1,833	8.2	8.3	3.3	343
	Day 2	27	95	1,504	8.2	35.6	6.0	416
	Day 3	27	83	814	8.0	4.0	1.7	229
	Day 7	28	97	922	8.5	4.2	2.1	206
	Day 14	29	78	4,218	8.0	4.4	0.3	nd
	Hour 0	28	95	1,492	8.0	0.1	0.1	206
Reference	Day 1	27	87	1,899	8.2	0.1	0.0	201
	Day 14	29	79	4,289	8.0	nd	nd	nd

^*a*^ nd, no data.

**Fig 3:**
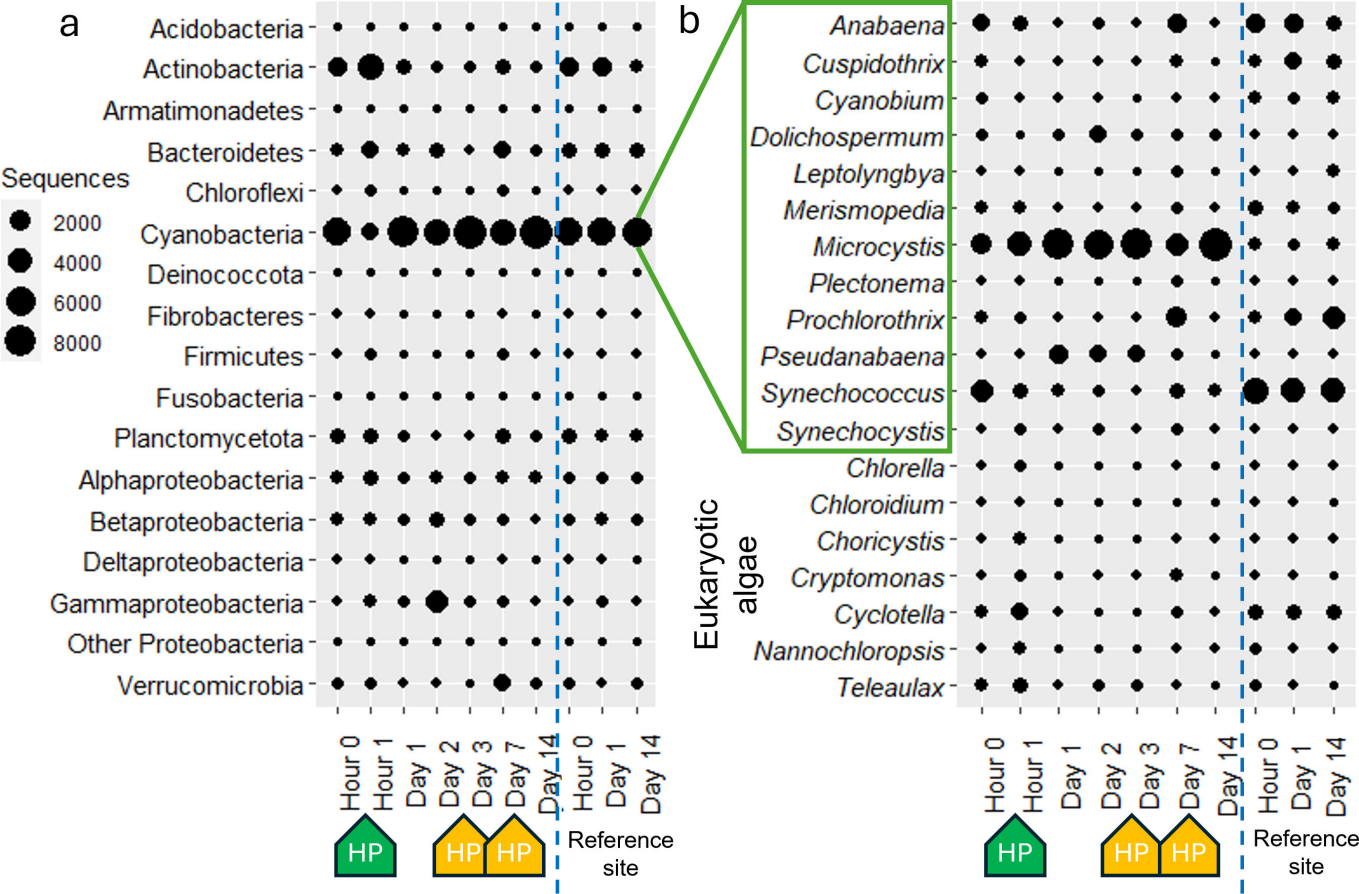
Bubble plots of high-throughput amplicon sequencing analyses of planktonic communities. (**a**) Bacterial phyla and (**b**) algal genera (cyanobacteria and eukaryotic algae). Major taxa (> 1%) are shown after normalization of sequence reads (per 10,000 reads). The bottom HP markers show liquid hydrogen peroxide (green) and Lake Guard Oxy (yellow) treatments.

Despite the promising response measured within 1 hour of treatment, the *Microcystis* bloom was not controlled from day 1 onward. Chl-a (477 µg/L) and algal colony density (8.3 × 10^5^ cells/L) rapidly increased by 113% and 1,713%, respectively, compared to hour 0. This likely reflects the rapid degradation and dilution of hydrogen peroxide in our open treatment area, which was insufficient to control the bloom beyond day 0. Cyanobacteria also increased to 78.1% of the bacterial community in amplicon sequencing data and fluctuated between 50.7% and 91.0% for the remainder of the experiment, while *Microcystis* remained dominant in the algal community amplicon sequencing data (Fig. 3; Table S1). On Day 2, the highest measurements of Chl-a concentration (2,076 µg/L), algal colony density (1.9 × 10^6^ cells/L), and microcystin concentration (total microcystin, 35.6 µg/L, extracellular microcystin 6.0 µg/L) of the entire experiment were observed (Fig. 2). The relative abundance of *Microcystis* also increased on day 2 from 22.5% to 65.0% within the algal community (Fig. 3). As a consequence, TP increased from 131 to 1,890 µg/L and TN increased from 5.6 to 34.1 mg/L. This coincided with our highest measure of NH_4_^+^ (64 µg/L), which increased throughout the experiment, from 16 µg/L to 57 µg/L before decreasing to 32 µg/L on day 14, while the control site at the mouth of the semi-enclosed treatment area did not show any notable change (Fig. 2). The lack of nutrient data from the control site for every sampling day hinders our ability to accurately deduce why nutrients increased between the first and second treatments. Past studies have observed nutrient pool increases associated with ruptured cyanobacterial cells affected by treatment (7, 12, 22); however, our algal data seem to contradict this (Fig. 2). The semi-enclosed nature of our field treatment area allows for the possibility of nutrient input into the river through advection or mixing. Specific conductivity data indicate that substantial water exchange occurred during our field experiment, with values changing from 1,504 to 814 µS/cm between days 2 and 3 and from 922 to 4,218 µS/cm between day 7 and day 14 (no measurements were taken between day 3 and 7 or between days 7 and 14). Therefore, water mass advection may have contributed to the observed results, including changes in algal abundance, community succession, and other environmental factors (Table 1). This highlights the challenge of treating cHABs in riverine systems, even in strategically selected areas such as our study site. Nonetheless, reporting the overall impact of the treatments provides valuable information.

**Fig 4:**
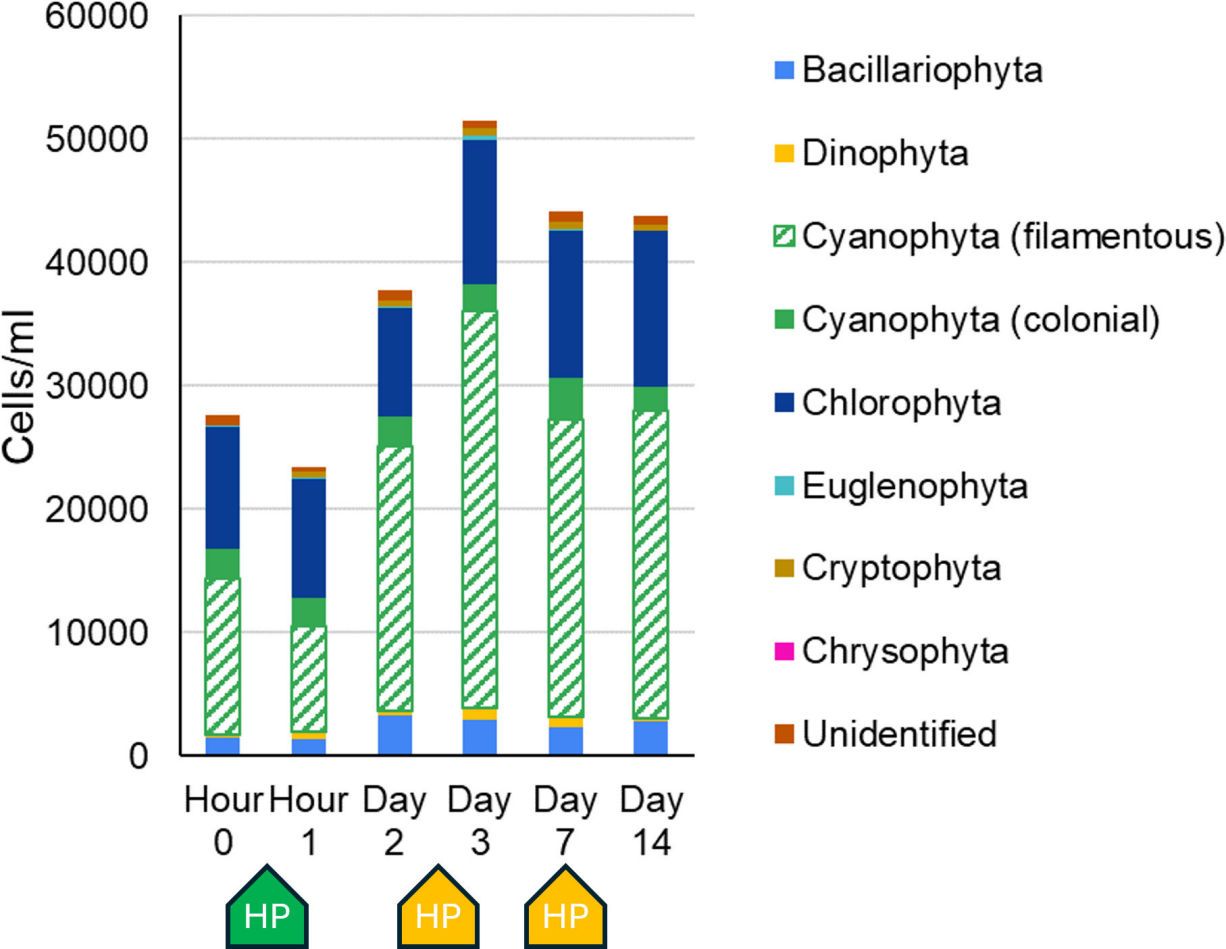
Phytoplankton counts were determined via microscopy identification. Colonial cyanobacteria during cHAB events were underestimated due to the floating nature of *Microcystis* cells. Picocyanobacteria were not counted by this method.

The Lake Guard Oxy application on day 7 successfully reduced Chl-a and algal colony density by 89.9% and 97.2% from day 3, respectively. Following the treatment, the total microcystin concentration remained stable, ranging from 4.0 to 4.2 µg/L, while the proportion of extracellular microcystin increased from 43% to 50% (Table 1). By day 7, cyanobacterial sequences had decreased by 46% compared to day 3 (Fig. 3; Table S1), with a particularly strong suppression of *Microcystis*, whose relative abundance declined by 67.2% of total algal sequences. On day 7, *Microcystis* dominance was replaced by two filamentous genera, one non-diazotrophic (*Prochlorothrix* [20.7%]) and one diazotrophic (*Anabaena* [16.9%]). The increase of filamentous cyanobacteria abundance was confirmed by phytoplankton microscopic counts (Fig. 4).

Subsequent repeated treatments with Lake Guard Oxy reduced the cHAB with Chl-a concentration (447 µg/L) and algal colony density (9.1 × 10^5^ cells/L) on day 14 being lower than the levels recorded between the liquid hydrogen peroxide application and the first Lake Guard Oxy treatment (days 1 and 2; Fig. 2). This may indicate Lake Guard Oxy was effective over multiple days after treatments, but within 1 week after application under these conditions, cHABs returned either via regrowth or advection. However, the lack of sample data between days 7 and 14 of our experiment does not allow us to draw strong conclusions about the interim effects of this treatment between days 7 and 14.

In the previous field hydrogen peroxide treatment study conducted at the same location in Florida, *Synechococcus* replaced the dominance of *Microcystis* after the treatment (12). In the present study, *Synechococcus* was the second-most abundant phytoplankton group as determined by amplicon sequencing data (Fig. 3b). Picocyanobacterial cell counting data showed a 19.7% decrease after the liquid treatment and a 32% decrease after Lake Guard Oxy (Fig. 2e). Phytoplankton sequencing data showed that *Synechococcus* declined from 30.2% to 8.8% 1 hour after the liquid hydrogen peroxide treatment and further decreased to 3.1% by the following day. *Synechococcus* continued to decline throughout the experiment, only partially recovering to 7.8% of phytoplankton sequences by day 7. Thus, strong conclusions about the effect of Lake Guard Oxy on *Synechococcus* cannot be drawn. However, cell count data suggest that the treatment may have been responsible for the decline of this nontarget cyanobacteria (Fig. 2e).

### Eukaryotic algal response

Eukaryotic algae represented a minor fraction of the phytoplankton community amplicon sequencing during the experiment at 4.9 ± 5.3% in the treatment area and 2.2 ± 1.2% at the reference site (Table S1). Interestingly, the highest relative abundance of eukaryotic algal sequences within the algal community during the experiment was 15.1%, observed 1 hour after the initial liquid hydrogen peroxide treatment. This represents a 217% increase from the 7.0% algal community representation measured before treatment, likely due to the selective removal of cyanobacterial fraction (Fig. 3). A similar effect was observed after Lake Guard Oxy application on day 7, with values increasing from 1.8% on day 3 to 5.1%. However, there was a difference in genera between the two treatment types. The genus *Teleaulax* was most abundant after the liquid treatment, contributing to 52.2% of eukaryotic algal sequences, and *Cryptomonas* was most abundant after Lake Guard Oxy, at 75.2% of eukaryotic algal sequences. Both genera are cryptophytes, and previous laboratory and field studies have shown that they are resilient to hydrogen peroxide treatments (5, 23).

### Transcriptomic analysis

**Fig 5:**
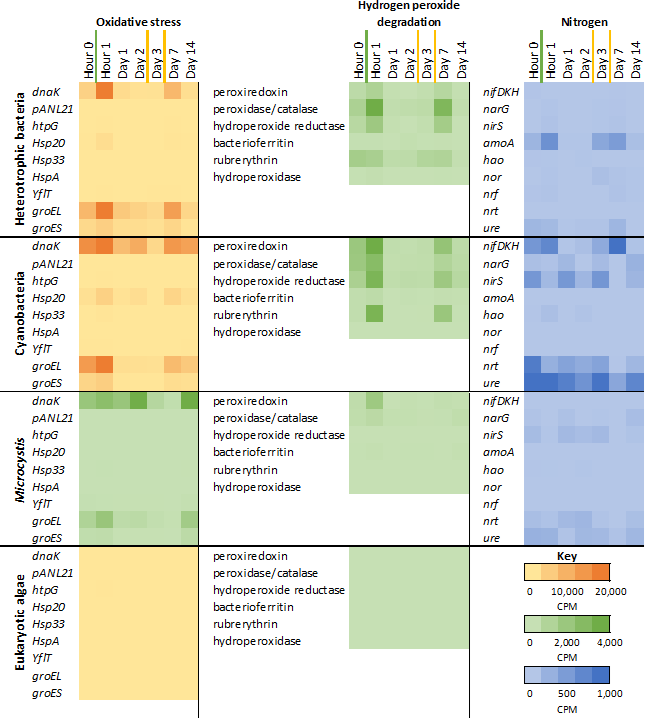
Heatmaps of oxidative stress, hydrogen peroxide degradation, and nitrogen-related gene expressions detected for heterotrophic bacteria, cyanobacteria, *Microcystis*, and eukaryotic phytoplankton. Different heatmap scales (denoted by color and listed in the figure key) have been used to show appropriate resolutions of expressions discussed. Liquid hydrogen peroxide spray (green line) and Lake Guard Oxy granular (yellow line) treatment additions denoted as lines between time labels. It should be noted that analysis of heterotrophic bacteria includes chemolithotrophic bacteria.

As shown in Fig. 5, transcriptomic data supported initial treatment effects for cyanobacteria, with upregulation of three oxidative stress genes for both liquid hydrogen peroxide (hour 1 compared to hour 0: fold-change upregulation: *htpG*: 2.5; *Hsp20*: 3.1; and *Hsp33*: 8.7) and Lake Guard Oxy (Day 7 compared to day 2: *htpG* increase from 0; fold-change upregulation: *Hsp20*: 1.9 and *Hsp33*: 5.1) (24, 25). There was also upregulation of the stress gene *groEL* (26) in *Microcystis* after the liquid hydrogen peroxide treatment (1.9 fold-change upregulation). It is important to note that the *groEL* and *dnaK* genes respond to a variety of stress conditions, but in this study, we interpret their response as oxidative stress because it coincided with our hydrogen peroxide treatments. Contrary to the greater effectiveness of Lake Guard Oxy indicated by our Chl-a and algal colony density data, there was no upregulation of oxidative stress genes in *Microcystis* shortly after Lake Guard Oxy treatments, neither on day 3 (1 day after a treatment) or on day 7 (1 hr after treatment). Regarding the rest of the bacterial community, results were mixed. No notable oxidative stress responses were observed on day 3; however, by day 7, both heterotrophic bacteria and cyanobacteria showed increased expression of *dnaK* (fold-change upregulation of 8.2 and 1.3, respectively) and *groEL* (fold-change upregulation of 3.4 and 5.7, respectively). This may indicate that Lake Guard Oxy was unable to sustain sufficient hydrogen peroxide levels to trigger a prolonged oxidative stress response as the day 3 sample was collected 1 day after application and the day 7 sample was taken 1 hour after application.

The degradation of hydrogen peroxide in aquatic systems is mediated in part by microbial enzymatic activity. Key enzymes involved in hydrogen peroxide detoxification include peroxiredoxins, peroxidases/catalases, hydroperoxide reductases, bacterioferritins, rubrerythrins, and hydroperoxidases, which help mitigate oxidative stress in microbial cells. Here, we assessed the temporal expression of these enzymes in heterotrophic bacteria, cyanobacteria, and eukaryotic algae following hydrogen peroxide exposure. At hour 1 (fold-change relative to hour 0) as well as day 3 and day 7 (fold-change relative to day 2), both heterotrophic bacteria and cyanobacteria exhibited upregulation of peroxiredoxin, peroxidase, hydroperoxide reductase, and rubrerythrin. These expression trends from heterotrophic bacteria and cyanobacteria were similar to results observed in our previous field application study at this site (12). *Microcystis* only had upregulation of peroxiredoxin and peroxidase at hour 0 with upregulated fold-changes of 5.2 and 1.9, respectively, and only peroxiredoxin on day 7 (fold-change upregulation of 1.6).

Among nitrogen-related genes, those associated with nitrogen fixation exhibited the most pronounced response to hydrogen peroxide treatments. A minor increase in *nifDHK* gene expression was observed between hour 0 and hour 1, followed by a major decrease between hour 1 and day 1. Both diazotrophs, Anabaena and Dolichospermum, decreased after the liquid hydrogen peroxide treatment. The *nifDKH* genes showed a 3.8-fold upregulation in transcription on day 3 (1 day after treatment) compared to day 2 before treatment. On day 7, as *Microcystis* dominance declined and was partially replaced by *Anabaena* and *Dolichospermum*, the nitrogen-fixation gene cluster *nifDKH* exhibited a 42.1-fold upregulation relative to day 2, before the Lake Guard Oxy treatment. These responses could be linked to the release of inorganic carbon (CO_3_^2-^/HCO_3_^-^) resulting from the degradation of sodium percarbonate into hydrogen peroxide and sodium carbonate. The additional inorganic carbon could serve as a readily available carbon source for diazotrophs, potentially supporting their metabolic activity and enhancing nitrogen fixation (27).

### Conclusion 

This study compared two different hydrogen peroxide-based treatments during a persistent *Microcystis* bloom at Franklin Lock and Dam in the Caloosahatchee River, Florida, USA. Both were effective in reducing cyanobacteria in the short term (right after the treatment to the next day) but failed over a longer timeframe (days to a week) perhaps due to water exchange in this flowing system. Lake Guard Oxy exhibited a more targeted effect on *Microcystis* removal, likely due to the tendency of *Microcystis* to form surface blooms and the buoyant nature of Lake Guard Oxy, which allows it to remain near the water surface. We also found that the Lake Guard Oxy treatment increased the gene expression of nitrogen fixation in filamentous diazotrophic genera such as *Anabaena* and *Dolichospermum*, which may stem from the release of percarbonate from Lake Guard Oxy. Needless to say, direct observation of nitrogen fixation via enzymatic activity is preferred to confidently draw conclusions beyond those based solely on observable transcriptomic succession. Overall, three treatments (one liquid and two Lake Guard Oxy) did not fully eliminate the bloom within 14 days, highlighting the limitations of hydrogen peroxide in open or flowing waters. These findings highlight the variability and complexity of hydrogen peroxide applications in natural water bodies, emphasizing the need for adaptive management strategies that account for environmental conditions and the properties of different treatment methods. Alternatively, a combination of other treatment techniques such as L-lysine may enhance the effectiveness of hydrogen peroxide treatments (28, 29). The research presented here contributes to the growing body of knowledge regarding potentially useful cHAB mitigation approaches and underscores the necessity for ongoing evaluation of treatment efficacy in diverse aquatic ecosystems.

## MATERIALS AND METHODS

### Study area

Franklin Lock and Dam at the Caloosahatchee River in Lee County, Florida, was selected as our study site. The Franklin Lock and Dam structure (S-79) separates the downstream tidally influenced (Gulf of Mexico) and upstream freshwater portions of the Caloosahatchee River. The river experienced tidal action twice per day (two low tides, two high tides) and receives freshwater input via rainfall and run-off, as well as controlled releases from Lake Okeechobee upstream, which is further gated by a subsequent series of locks (Fig. S1). Our site received no rainfall during our study period, but discharge from the S-79 lock structure (recorded at 15 min intervals by USGS: waterdata.usgs.gov) ranged from 4 to 88 cubic meters per second. The experiment was conducted on the downstream side of the lock structure in a 400 m² area (8 m × 50 m), oriented perpendicular to the shoreline and parallel to the lock wall. The 160 m arm of the lock structure parallel to shore offers a semi-enclosed space that is sheltered by the river’s flow, with only a roughly 50 m wide downstream-facing mouth open to the rest of the river (Fig. 1). However, our specific conductivity data indicate water exchange occurred (Table 1), influenced by both tidal flux and discharge from the S-79 lock.

### Hydrogen peroxide treatments

Two different hydrogen peroxide treatment methods were used in this field study: liquid hydrogen peroxide applied by FGCU and granular Lake Guard Oxy applied by BlueGreen Water Technologies. The latter is composed of 2% (wt/wt) inert biodegradable encapsulating agent that floats and time-releases the remaining 98% (wt/wt) sodium percarbonate, which releases hydrogen peroxide at the water surface.

Over the course of the study, three treatments occurred. At hour 0, 12 L of 3% hydrogen peroxide solution was directly applied via a 1-gallon hand pump sprayer from kayak for a final theoretical concentration of 20 mg/L (580 µM, 0.0018%) calculated based on a dilution depth of 10 cm across the 400 m^2^ treatment area. On days 2 (after the day 2 sample) and on day 7 (1 hour before sampling), 65 kg (143.3 pounds) of Lake Guard Oxy was used across a 2,023 m^2^ (0.5 acres) area (Fig. 1). This concentration is equivalent to the maximum single treatment dose of Lake Guard Oxy provided on the label approved by the US EPA, which is recommended to use when Chl-a concentration exceeds 50 µg/L and cyanobacterial cell density is more than 10^5^ cells/L. This multiple treatment scheme was the result of multiple organizations working in the same area. What started as a liquid hydrogen peroxide field application study morphed into the opportunity to examine both liquid and granular hydrogen peroxide-based treatments in concert.

### Hydrogen peroxide analysis

A total of three 10 mL replicate samples were collected from three points equally spaced across the length of the treatment area for hydrogen peroxide measurements. Each replicate was independently and immediately filtered through 0.2 µm Sterivex filters. Filtered water was frozen in a sealed container of liquid nitrogen on site and transported to a −80°C freezer within a few hours until analyzed to prevent degradation. Hydrogen peroxide was measured with a fast response amperometric 250 µm diameter hydrogen peroxide microelectrode with a built-in reference electrode (HP-250, Innovative Instruments, Tampa, FL, USA) featuring a lower detection limit of 50 nM 1.7 µg/L using methods described in (8).

### Field sampling

Nutrients (TP: USEPA method 365.2; OP: USEPA method 365.3; NH_4_^+^: USEPA method 350.1; TKN: USEPA method 351.2; NOx: Systea Easy Standard Method) and chlorophyll-a (Chl-a) (USEPA method 445) were measured by a National Environmental Laboratories Accreditation Conference-approved laboratory (NELAC) (Benchmark EnviroAnalytical Inc., FL, USA) within 48 h of sample collection.

Microcystin samples were handled and analyzed via USEPA method 546 and manufacturer directions using an enzyme-linked immunosorbent assay (ELISA) (Beacon Analytic Systems Inc., ME, USA). To differentiate total and extracellular microcystin, unfiltered and 0.22 µm filtered subsamples were prepared and analyzed.

Samples for bacterial and picocyanobacterial cell enumeration were preserved with formalin (2% (vol/vol) as the final concentration) and stored at −80°C. Fixed water samples were stained with SYBR Green I for 5 min and collected onto a 0.2 µm pore-sized black polycarbonate filter (25 mm, MilliporeSigma). The anti-bleaching agent Vectashield (Vector Laboratories, CA, USA) was used as a mounting medium. Cells were viewed with an Olympus BX-51 epifluorescence microscope (Tokyo, Japan). Algal colony counting and counting and identification of phytoplankton cells were conducted using methods previously described by Hancock et al. (16).

### 16S rRNA gene amplicon sequencing

Microbial cells were collected by filtering 200 mL of water with a 0.22 µm cellulose nitrate filter. Genomic DNA was extracted from half-cut filter samples using the DNeasy PowerSoil Pro Kit (Qiagen, MD, USA) following the manufacturer’s instructions. High-throughput amplicon sequencing was conducted using the Illumina MiSeq system (MR DNA, Shallowater, TX, USA). For bacteria, the V4–V5 region of the 16S rRNA gene was amplified using the primer pair 515yF and 926pfR (30). For photosynthetic microeukaryotes and cyanobacteria, the chloroplast 16S rRNA gene was targeted using the primary pair 359F and 781R (V3–V4 region) (31). While this primer pair captures the 16S rRNA gene of cyanobacteria, it also amplifies plastid 16S rRNA genes from photosynthetic eukaryotes. A 30-cycle PCR using the HotStarTaq Plus Master Mix Kit (Qiagen, MD, USA) was run under the following conditions: 95°C for 5 min, followed by 30 cycles of 95°C for 30 sec, 53°C for 40 sec, and 72°C for 1 min, after which a final elongation step at 72°C for 10 min was performed. After amplification, PCR products were checked in 2% agarose gel. Samples were multiplexed using unique dual indices and pooled together in equal proportions based on their molecular weight and DNA concentrations. Pooled samples were purified using calibrated Ampure XP beads (Beckman Coulter Life Sciences, IN, US). Then, the pooled and purified PCR product was used to prepare an Illumina DNA library. For analysis, sequences less than 150 base pairs (bp) and containing ambiguous base calls were removed. The dereplicated or unique sequences were denoised; unique sequences identified with sequencing and/or PCR point errors were removed, followed by chimera removal, thereby providing a denoised sequence or amplicon sequence variant (ASV). Final ASVs were taxonomically classified to genus via representative ASVs through BLAST at the NCBI. Samples underwent normalization (scaled to 10,000 reads) before further analysis.

### Metatranscriptome sequencing

RNA samples for metatranscriptome sequencing were collected by filtering 200 mL of water using 0.2 µm Sterivex filters on-site that were promptly frozen in liquid nitrogen for transport to a −80°C freezer until further analysis. Total RNA was isolated from Sterivex filters using the RNeasy PowerMicrobiome Kit following the manufacturer’s instructions (Qiagen, MD, USA). For each filter sample, three extractions were performed. RNA was eluted in 100 µL RNase-free water and pooled. DNA contamination was removed using Baseline-ZERO DNase (Epicenter, CA, USA) following the manufacturer’s instructions followed by purification using RNA Clean & Concentrator columns (Zymo Research, CA, USA). The RNA samples were used for rRNA removal by using Ribo-Zero Plus rRNA Depletion Kit (Illumina, CA, USA). rRNA-depleted samples were used for library preparation via the KAPA mRNA HyperPrep Kits (Roche, Basel, Switzerland) by following the manufacturer’s instructions. The average library size was determined using the Agilent 2100 Bioanalyzer (Agilent Technologies, CA, USA), and the sample was then pooled in equimolar ratios of 0.6 nM and sequenced paired-end for 300 bases using the NovaSeq 6000 system (Illumina). The amplified samples were used for library preparation using the Illumina DNA Prep (M) Tagmentation library preparation kit (Illumina) following the manufacturer’s user guide. Resulting data were processed and analyzed via the MG-RAST pipeline (32) for taxonomy and function with the following parameters: 10^−5^ e value, 60% identity, and a minimum alignment length of 16 amino acids. Functional analysis was performed using NCBI RefSeq (US National Center for Biotechnology Information Reference Sequence Database) protein annotation. Using the edgeR package in R (33), transcripts were normalized by the trimmed means of M-values (TMM) to account for library size differences, and then count per million (CPM) was used for gene expression analyses. Specific genes of interest were examined within specific taxonomic groups of interest, and fold change was calculated relative to the total number of transcripts of the group. No integration of the metatranscriptomic and amplicon sequencing data was performed. Functional and taxonomic assignments were based solely on MG-RAST pipeline analysis.

### Highlights

- Among three sequenced treatments, short time reductions of cyanobacteria were observed.
- Different modes of action were observed between liquid H_2_O_2_ and slow-release Lake Guard Oxy.
- Despite three peroxide-based treatments, algal biomass persisted after 2 weeks.
- Lake Guard Oxy stimulated the expression of nitrogen fixation genes in filamentous cyanobacteria.

## Data Availability

Raw sequence data were deposited to the NCBI Sequence Read Archive (SRA) via BioProject accession number PRJNA1244001.

## SUPPLEMENTAL MATERIAL

Supplemental material aem.01950-25-s0001.docx Table S1 and Fig. S1.

ASM does not own the copyrights to Supplemental Material that may be linked to, or accessed through, an article. The authors have granted ASM a non-exclusive, world-wide license to publish the Supplemental Material files. Please contact the corresponding author directly for reuse.
