# Supplementary material for: Microcystis bloom control using hydrogen peroxide and floating sodium percarbonate algaecide Lake Guard Oxy in Florida
Source: Appl Environ Microbiol. 2025 Dec 2;91(12):e01950-25. doi: 10.1128/aem.01950-25 (PMC12724209; doi:10.1128/aem.01950-25)
Supplement: Supplemental material — Table S1 and Fig. S1. [file aem.01950-25-s0001.docx]

**Table S1.** Normalized sequence reads (SR) and relative abundance (RA; rounded to the nearest 0.1%) of bacterial and algal amplicon sequencing.

|  |  |  |  |  |  |  |  |  |  |  |  |  |  |  |  |  |  |  |  |  |  |
| --- | --- | --- | --- | --- | --- | --- | --- | --- | --- | --- | --- | --- | --- | --- | --- | --- | --- | --- | --- | --- | --- |
|  | Bacterial | Hour 0 |  | Hour 1 |  | Day 1 |  | Day 2 |  | Day 3 |  | Day 7 |  | Day 14 |  | Hour 0 |  | Day 1 |  | Day 14 |  |
|  | phylum | SR* | RA† | SR | RA | SR | RA | SR | RA | SR | RA | SR | RA | SR | RA | SR | RA | SR | RA | SR | RA |
|  | Acidobacteria | 9 | (0.1%) | 14 | (0.1%) | 3 | (0%) | 2 | (0%) | 1 | (0%) | 2 | (0%) | 0 | (0%) | 9 | (0.1%) | 5 | (0.1%) | 3 | (0%) |
|  | Actinobacteria | 1547 | (15.5%) | 4230 | (42.3%) | 696 | (7%) | 137 | (1.4%) | 127 | (1.3%) | 827 | (8.3%) | 158 | (1.6%) | 1732 | (17.3%) | 1930 | (19.3%) | 594 | (5.9%) |
|  | Alphaproteobacteria | 324 | (3.2%) | 667 | (6.7%) | 292 | (2.9%) | 396 | (4%) | 243 | (2.4%) | 370 | (3.7%) | 427 | (4.3%) | 307 | (3.1%) | 285 | (2.9%) | 270 | (2.7%) |
|  | Armatimonadetes | 17 | (0.2%) | 17 | (0.2%) | 4 | (0%) | 1 | (0%) | 0 | (0%) | 13 | (0.1%) | 3 | (0%) | 15 | (0.2%) | 13 | (0.1%) | 10 | (0.1%) |
|  | Bacteroidetes | 599 | (6%) | 1273 | (12.7%) | 338 | (3.4%) | 738 | (7.4%) | 100 | (1%) | 1091 | (10.9%) | 173 | (1.7%) | 611 | (6.1%) | 697 | (7%) | 664 | (6.6%) |
|  | Betaproteobacteria | 362 | (3.6%) | 572 | (5.7%) | 292 | (2.9%) | 701 | (7%) | 165 | (1.7%) | 318 | (3.2%) | 93 | (0.9%) | 308 | (3.1%) | 371 | (3.7%) | 270 | (2.7%) |
|  | Chloroflexi | 62 | (0.6%) | 139 | (1.4%) | 13 | (0.1%) | 7 | (0.1%) | 7 | (0.1%) | 162 | (1.6%) | 11 | (0.1%) | 58 | (0.6%) | 57 | (0.6%) | 93 | (0.9%) |
|  | Cyanobacteria | 5936 | (59.4%) | 1246 | (12.5%) | 7811 | (78.1%) | 5067 | (50.7%) | 9105 | (91.1%) | 4917 | (49.2%) | 8601 | (86%) | 5849 | (58.5%) | 5706 | (57.1%) | 7108 | (71.1%) |
|  | Deinococcota | 8 | (0.1%) | 18 | (0.2%) | 3 | (0%) | 3 | (0%) | 1 | (0%) | 11 | (0.1%) | 1 | (0%) | 5 | (0.1%) | 5 | (0.1%) | 1 | (0%) |
|  | Deltaproteobacteria | 34 | (0.3%) | 72 | (0.7%) | 11 | (0.1%) | 7 | (0.1%) | 1 | (0%) | 59 | (0.6%) | 4 | (0%) | 35 | (0.4%) | 36 | (0.4%) | 19 | (0.2%) |
|  | Fibrobacteres | 59 | (0.6%) | 112 | (1.1%) | 15 | (0.2%) | 6 | (0.1%) | 4 | (0%) | 78 | (0.8%) | 4 | (0%) | 77 | (0.8%) | 47 | (0.5%) | 58 | (0.6%) |
|  | Firmicutes | 80 | (0.8%) | 143 | (1.4%) | 20 | (0.2%) | 19 | (0.2%) | 9 | (0.1%) | 127 | (1.3%) | 21 | (0.2%) | 87 | (0.9%) | 66 | (0.7%) | 50 | (0.5%) |
|  | Fusobacteria | 5 | (0.1%) | 11 | (0.1%) | 12 | (0.1%) | 11 | (0.1%) | 5 | (0.1%) | 6 | (0.1%) | 5 | (0.1%) | 2 | (0%) | 3 | (0%) | 4 | (0%) |
|  | Gammaproteobacteria | 111 | (1.1%) | 376 | (3.8%) | 264 | (2.6%) | 2700 | (27%) | 144 | (1.4%) | 129 | (1.3%) | 69 | (0.7%) | 104 | (1%) | 159 | (1.6%) | 91 | (0.9%) |
|  | Other Proteobacteria | 3 | (0%) | 17 | (0.2%) | 7 | (0.1%) | 8 | (0.1%) | 3 | (0%) | 0 | (0%) | 10 | (0.1%) | 3 | (0%) | 6 | (0.1%) | 2 | (0%) |
|  | Planctomycetota | 652 | (6.5%) | 899 | (9%) | 166 | (1.7%) | 124 | (1.2%) | 64 | (0.6%) | 721 | (7.2%) | 282 | (2.8%) | 620 | (6.2%) | 504 | (5%) | 515 | (5.2%) |
|  | Verrucomicrobia | 192 | (1.9%) | 196 | (2%) | 50 | (0.5%) | 73 | (0.7%) | 20 | (0.2%) | 1169 | (11.7%) | 136 | (1.4%) | 178 | (1.8%) | 110 | (1.1%) | 250 | (2.5%) |
|  |  |  |  |  |  |  |  |  |  |  |  |  |  |  |  |  |  |  |  |  |  |
| Algal |  | Hour 0 |  | Hour 1 |  | Day 1 |  | Day 2 |  | Day 3 |  | Day 7 |  | Day 14 |  | Hour 0 |  | Day 1 |  | Day 14 |  |
| group | Genus | SR | RA | SR | RA | SR | RA | SR | RA | SR | RA | SR | RA | SR | RA | SR | RA | SR | RA | SR | RA |
| Bacillariophyta | *Aulacoseira* | 32 | (0.3%) | 93 | (0.9%) | 0 | (0%) | 0 | (0%) | 1 | (0%) | 21 | (0.2%) | 1 | (0%) | 32 | (0.3%) | 41 | (0.4%) | 17 | (0.2%) |
| Bacillariophyta | *Cyclotella* | 492 | (4.9%) | 1089 | (10.9%) | 26 | (0.3%) | 8 | (0.1%) | 10 | (0.1%) | 259 | (2.6%) | 25 | (0.3%) | 672 | (6.7%) | 815 | (8.2%) | 730 | (7.3%) |
| Bacillariophyta | *Stephanodiscus* | 8 | (0.1%) | 26 | (0.3%) | 0 | (0%) | 0 | (0%) | 0 | (0%) | 2 | (0%) | 0 | (0%) | 7 | (0.1%) | 13 | (0.1%) | 4 | (0%) |
| Bacillariophyta | *Triceratium* | 39 | (0.4%) | 77 | (0.8%) | 14 | (0.1%) | 6 | (0.1%) | 7 | (0.1%) | 25 | (0.3%) | 22 | (0.2%) | 17 | (0.2%) | 28 | (0.3%) | 39 | (0.4%) |
| Chlorophyta | *Acutodesmus* | 1 | (0%) | 5 | (0.1%) | 0 | (0%) | 0 | (0%) | 0 | (0%) | 1 | (0%) | 0 | (0%) | 3 | (0%) | 0 | (0%) | 1 | (0%) |
| Chlorophyta | *Chlorella* | 44 | (0.4%) | 202 | (2%) | 4 | (0%) | 3 | (0%) | 3 | (0%) | 40 | (0.4%) | 6 | (0.1%) | 55 | (0.6%) | 51 | (0.5%) | 54 | (0.5%) |
| Chlorophyta | *Chloroidium* | 47 | (0.5%) | 116 | (1.2%) | 3 | (0%) | 4 | (0%) | 3 | (0%) | 19 | (0.2%) | 1 | (0%) | 70 | (0.7%) | 33 | (0.3%) | 11 | (0.1%) |
| Chlorophyta | *Choricystis* | 109 | (1.1%) | 333 | (3.3%) | 12 | (0.1%) | 7 | (0.1%) | 5 | (0.1%) | 33 | (0.3%) | 33 | (0.3%) | 118 | (1.2%) | 82 | (0.8%) | 39 | (0.4%) |
| Chlorophyta | *Mychonastes* | 10 | (0.1%) | 21 | (0.2%) | 0 | (0%) | 0 | (0%) | 1 | (0%) | 7 | (0.1%) | 0 | (0%) | 13 | (0.1%) | 5 | (0.1%) | 2 | (0%) |
| Chlorophyta | *Nephroselmis* | 6 | (0.1%) | 14 | (0.1%) | 0 | (0%) | 0 | (0%) | 1 | (0%) | 1 | (0%) | 2 | (0%) | 5 | (0.1%) | 1 | (0%) | 9 | (0.1%) |
| Chlorophyta | *Pectinodesmus* | 2 | (0%) | 7 | (0.1%) | 1 | (0%) | 0 | (0%) | 0 | (0%) | 5 | (0.1%) | 1 | (0%) | 1 | (0%) | 2 | (0%) | 8 | (0.1%) |
| Chlorophyta | *Picochlorum* | 15 | (0.2%) | 68 | (0.7%) | 2 | (0%) | 1 | (0%) | 1 | (0%) | 15 | (0.2%) | 0 | (0%) | 22 | (0.2%) | 10 | (0.1%) | 5 | (0.1%) |
| Chlorophyta | *Tetrabaena* | 2 | (0%) | 4 | (0%) | 0 | (0%) | 0 | (0%) | 0 | (0%) | 6 | (0.1%) | 1 | (0%) | 3 | (0%) | 1 | (0%) | 2 | (0%) |
| Cyanobacteria | *Anabaena* | 1223 | (12.2%) | 806 | (8.1%) | 99 | (1%) | 295 | (3%) | 85 | (0.9%) | 1685 | (16.9%) | 21 | (0.2%) | 1487 | (14.9%) | 1598 | (16%) | 778 | (7.8%) |
| Cyanobacteria | *Aphanizomenon* | 1 | (0%) | 1 | (0%) | 3 | (0%) | 38 | (0.4%) | 5 | (0.1%) | 34 | (0.3%) | 3 | (0%) | 0 | (0%) | 1 | (0%) | 7 | (0.1%) |
| Cyanobacteria | *Cuspidothrix* | 333 | (3.3%) | 103 | (1%) | 27 | (0.3%) | 46 | (0.5%) | 27 | (0.3%) | 471 | (4.7%) | 7 | (0.1%) | 382 | (3.8%) | 1205 | (12.1%) | 663 | (6.6%) |
| Cyanobacteria | *Cyanobium* | 270 | (2.7%) | 86 | (0.9%) | 30 | (0.3%) | 23 | (0.2%) | 11 | (0.1%) | 74 | (0.7%) | 118 | (1.2%) | 321 | (3.2%) | 224 | (2.2%) | 453 | (4.5%) |
| Cyanobacteria | *Cylindrospermopsis* | 1 | (0%) | 21 | (0.2%) | 0 | (0%) | 0 | (0%) | 1 | (0%) | 59 | (0.6%) | 0 | (0%) | 3 | (0%) | 6 | (0.1%) | 22 | (0.2%) |
| Cyanobacteria | *Dolichospermum* | 138 | (1.4%) | 7 | (0.1%) | 287 | (2.9%) | 1207 | (12.1%) | 279 | (2.8%) | 128 | (1.3%) | 206 | (2.1%) | 43 | (0.4%) | 64 | (0.6%) | 22 | (0.2%) |
| Cyanobacteria | *Gloeocapsa* | 8 | (0.1%) | 93 | (0.9%) | 1 | (0%) | 1 | (0%) | 0 | (0%) | 22 | (0.2%) | 1 | (0%) | 11 | (0.1%) | 17 | (0.2%) | 12 | (0.1%) |
| Cyanobacteria | *Gloeothece* | 3 | (0%) | 11 | (0.1%) | 0 | (0%) | 1 | (0%) | 0 | (0%) | 4 | (0%) | 0 | (0%) | 3 | (0%) | 8 | (0.1%) | 4 | (0%) |
| Cyanobacteria | *Halomicronema* | 2 | (0%) | 7 | (0.1%) | 0 | (0%) | 1 | (0%) | 2 | (0%) | 23 | (0.2%) | 1 | (0%) | 0 | (0%) | 0 | (0%) | 8 | (0.1%) |
| Cyanobacteria | *Leptolyngbya* | 35 | (0.4%) | 49 | (0.5%) | 3 | (0%) | 2 | (0%) | 4 | (0%) | 186 | (1.9%) | 8 | (0.1%) | 66 | (0.7%) | 76 | (0.8%) | 331 | (3.3%) |
| Cyanobacteria | *Limnococcus* | 7 | (0.1%) | 7 | (0.1%) | 0 | (0%) | 0 | (0%) | 0 | (0%) | 1 | (0%) | 0 | (0%) | 5 | (0.1%) | 1 | (0%) | 1 | (0%) |
| Cyanobacteria | *Merismopedia* | 573 | (5.7%) | 345 | (3.5%) | 63 | (0.6%) | 68 | (0.7%) | 27 | (0.3%) | 189 | (1.9%) | 74 | (0.7%) | 623 | (6.2%) | 355 | (3.6%) | 227 | (2.3%) |
| Cyanobacteria | *Microcystis* | 2247 | (22.5%) | 3532 | (35.3%) | 7122 | (71.2%) | 6498 | (65%) | 7889 | (78.9%) | 2594 | (25.9%) | 8714 | (87.1%) | 316 | (3.2%) | 306 | (3.1%) | 372 | (3.7%) |
| Cyanobacteria | *Nodosilinea* | 1 | (0%) | 1 | (0%) | 0 | (0%) | 0 | (0%) | 0 | (0%) | 26 | (0.3%) | 0 | (0%) | 1 | (0%) | 3 | (0%) | 9 | (0.1%) |
| Cyanobacteria | *Plectonema* | 42 | (0.4%) | 73 | (0.7%) | 9 | (0.1%) | 7 | (0.1%) | 10 | (0.1%) | 158 | (1.6%) | 14 | (0.1%) | 81 | (0.8%) | 78 | (0.8%) | 101 | (1%) |
| Cyanobacteria | *Prochlorococcus* | 24 | (0.2%) | 2 | (0%) | 1 | (0%) | 0 | (0%) | 0 | (0%) | 1 | (0%) | 1 | (0%) | 41 | (0.4%) | 70 | (0.7%) | 38 | (0.4%) |
| Cyanobacteria | *Prochlorothrix* | 357 | (3.6%) | 172 | (1.7%) | 77 | (0.8%) | 39 | (0.4%) | 56 | (0.6%) | 2067 | (20.7%) | 105 | (1.1%) | 559 | (5.6%) | 979 | (9.8%) | 2548 | (25.5%) |
| Cyanobacteria | *Pseudanabaena* | 105 | (1.1%) | 37 | (0.4%) | 1745 | (17.5%) | 1015 | (10.2%) | 1169 | (11.7%) | 208 | (2.1%) | 16 | (0.2%) | 38 | (0.4%) | 36 | (0.4%) | 32 | (0.3%) |
| Cyanobacteria | *Snowella* | 3 | (0%) | 8 | (0.1%) | 0 | (0%) | 0 | (0%) | 0 | (0%) | 6 | (0.1%) | 1 | (0%) | 3 | (0%) | 2 | (0%) | 3 | (0%) |
| Cyanobacteria | *Sphaerospermopsis* | 7 | (0.1%) | 7 | (0.1%) | 1 | (0%) | 4 | (0%) | 1 | (0%) | 6 | (0.1%) | 0 | (0%) | 4 | (0%) | 10 | (0.1%) | 2 | (0%) |
| Cyanobacteria | *Synechococcus* | 3025 | (30.3%) | 876 | (8.8%) | 314 | (3.1%) | 287 | (2.9%) | 108 | (1.1%) | 778 | (7.8%) | 468 | (4.7%) | 4590 | (45.9%) | 3648 | (36.5%) | 3270 | (32.7%) |
| Cyanobacteria | *Synechocystis* | 86 | (0.9%) | 160 | (1.6%) | 80 | (0.8%) | 251 | (2.5%) | 115 | (1.2%) | 123 | (1.2%) | 97 | (1%) | 33 | (0.3%) | 40 | (0.4%) | 32 | (0.3%) |
| Cyanobacteria | *Xenococcus* | 1 | (0%) | 3 | (0%) | 0 | (0%) | 0 | (0%) | 0 | (0%) | 20 | (0.2%) | 0 | (0%) | 2 | (0%) | 4 | (0%) | 21 | (0.2%) |
| Eukaryota | *Chroomonas* | 55 | (0.6%) | 93 | (0.9%) | 8 | (0.1%) | 8 | (0.1%) | 1 | (0%) | 7 | (0.1%) | 20 | (0.2%) | 22 | (0.2%) | 12 | (0.1%) | 58 | (0.6%) |
| Eukaryota | *Cryptomonas* | 98 | (1%) | 248 | (2.5%) | 18 | (0.2%) | 26 | (0.3%) | 43 | (0.4%) | 523 | (5.2%) | 1 | (0%) | 49 | (0.5%) | 34 | (0.3%) | 1 | (0%) |
| Eukaryota | *Proteomonas* | 10 | (0.1%) | 7 | (0.1%) | 0 | (0%) | 0 | (0%) | 0 | (0%) | 0 | (0%) | 1 | (0%) | 1 | (0%) | 0 | (0%) | 1 | (0%) |
| Eukaryota | *Teleaulax* | 422 | (4.2%) | 790 | (7.9%) | 36 | (0.4%) | 142 | (1.4%) | 130 | (1.3%) | 67 | (0.7%) | 6 | (0.1%) | 142 | (1.4%) | 56 | (0.6%) | 3 | (0%) |
| Eustigmatophyceae | *Nannochloropsis* | 102 | (1%) | 342 | (3.4%) | 10 | (0.1%) | 8 | (0.1%) | 6 | (0.1%) | 80 | (0.8%) | 21 | (0.2%) | 125 | (1.3%) | 65 | (0.7%) | 49 | (0.5%) |
| Rhodophyta | *Ceramium* | 1 | (0%) | 6 | (0.1%) | 1 | (0%) | 1 | (0%) | 0 | (0%) | 0 | (0%) | 0 | (0%) | 1 | (0%) | 1 | (0%) | 0 | (0%) |
| Streptophyta | *Closterium* | 2 | (0%) | 11 | (0.1%) | 0 | (0%) | 0 | (0%) | 0 | (0%) | 8 | (0.1%) | 0 | (0%) | 0 | (0%) | 4 | (0%) | 9 | (0.1%) |
| Streptophyta | *Interfilum* | 8 | (0.1%) | 17 | (0.2%) | 1 | (0%) | 2 | (0%) | 0 | (0%) | 9 | (0.1%) | 0 | (0%) | 21 | (0.2%) | 10 | (0.1%) | 1 | (0%) |

**
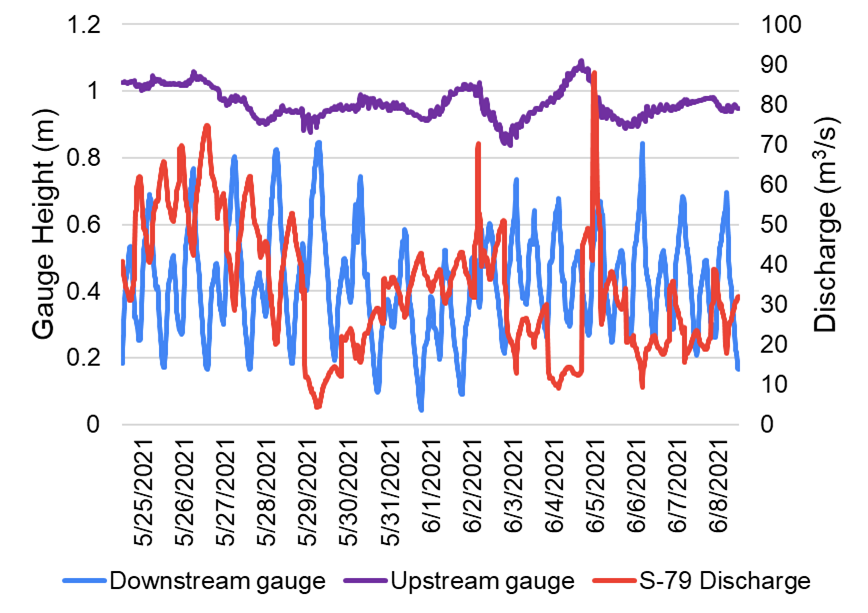
Figure S1.** Hydrology dynamics of the study site. Line graph showing upstream and downstream water levels and discharge rates through the S-79 lock structure. Data were obtained from the U.S. Geological Survey (USGS) at [waterdata.usgs.gov](https://waterdata.usgs.gov).
